# Individual, social and environmental determinants of smokeless tobacco and betel quid use amongst adolescents of Karachi: a school-based cross-sectional survey

**DOI:** 10.1186/s12889-017-4916-1

**Published:** 2017-11-28

**Authors:** Azmina Hussain, Sidra Zaheer, Kashif Shafique

**Affiliations:** 1 Dr. Ishrat-ul-Ibad Khan Institute of Oral Health Sciences, Dow University of Health Sciences, OJHA Campus, Suparco Road, Gulzar-e-Hijri, Scheme, Karachi, 33 Pakistan; 2 School of Public Health, Dow University of Health Sciences, OJHA Campus, SUPARCO road, Gulzar e Hijri, Karachi, Pakistan; 3 Institute of Health and Wellbeing, Public Health, University of Glasgow, 1-Lilybank Gardens, G12 8RZ, Glasgow, UK

**Keywords:** Smokeless tobacco, Betel quid, Adolescents, Social and environmental determinants

## Abstract

**Background:**

With 600 million people using betel quid (BQ) globally, and smokeless tobacco (SLT) use being more wide-spread; the duo is an uphill public health concern in South Asian countries. SLT and/or BQ use increases the risk for morbidity and mortality from oral cancer. Because SLT and/or BQ use is initiated during adolescence, it renders this group more vulnerable; and particular attention is needed to curb SLT and/or BQ use to reduce related disease burden. We aimed to observe the differential individual, social and environmental features amongst SLT and/or BQ users to determine the key influencers of its use in adolescents.

**Methods:**

This study was a cross-sectional survey of 2140 adolescents from secondary schools of Karachi, Pakistan. The main outcome measure was SLT and/or BQ use based on their consumption in the past 30 days. Univariate and multivariate regression binary logistic analyses were employed while reporting results in both crude form and adjusted odds ratio (after adjusting for all remaining individual, social and environmental level variables) with 95% confidence level. A *p*-value of < .05 was considered significant for all analyses.

**Results:**

The overall prevalence of SLT and/or BQ use was 42.6% (*n* = 912) of the total sample. The SLT and/or BQ consumer group had more males than females. A significant proportion of user (*n* = 558, 61.2%) was found in co-education schools. Students whose peers (OR = 6.79, 95% CI 4.67–9.87, *p*-value <0.001) and/or either of the parents (OR = 2.16, 95% CI 1.73–2.65, *p*-value <0.001) used SLT and/or BQ, alongside, adolescents who had not attended knowledge based sessions in schools regarding harmful effects of SLT and/or BQ were more likely to consume it. It’s availability with outside school hawkers increased the odds of its use by 6 times, as indicated by both univariate and multivariate models after adjusting for the remaining variables.

**Conclusion:**

In conclusion, students studying in co-education, parents and peers use, lack of knowledge based sessions on harmful health effects of SLT and/or BQ, and easy availability of the product from hawkers outside school all contribute towards enhanced risk of SLT and/or BQ use in adolescents.

## Background

Tobacco is amongst the leading evitable causes of global morbidity and mortality [1]. In contemporary times, nearly 6 million lives are lost due to tobacco consumption which is anticipated to rise exponentially and may hit 8 million by the year 2030 with its major burden in third world countries [2]. This will render tobacco as the major cause of deaths worldwide.

All various forms of tobacco have been associated with adverse health effects inclusive of oral cancer [3]. With 600 million people using betel quid (BQ) globally [4], and smokeless tobacco (SLT) use being more widely spread than ever [5]; the deadly combination is a constant uphill public health concern specially in South Asian countries in terms of increased morbidity and mortality from oral cancer [6].

Smokeless tobacco is the pre-dominant form of tobacco that is accountable for the most tobacco related ill health effects [7]. SLT contains nicotine and carcinogenic nitrosamines which cause more than a quarter million deaths mainly due to oral and pharyngeal cancers [3, 5].

Betel Quid (paan) with or without tobacco, areca nut (supari, chaalia), ghutka, niswar, tobacco smoking in all forms are established oral carcinogens by IARC [6, 8–10] which also cause oral potentially malignant lesions [4].

SLT consumption is quite acceptable in South Asian countries as part of their cultural and social norms [11]. At a very young age, individuals start consuming it as these products are readily available and considered as sweets [12]. A variety of factors are included and the list is not exhaustive to family values, easy access, cheap in cost, as dental pain relieving medicine, no enforcement of legislation on its use; are responsible factors for SLT wide spread use [13, 14]. The women and young individuals in India, Pakistan and Bangladesh refrain from smoking due to cultural and traditional beliefs but there is no such hesitation in SLT and/or BQ use; rather parents encourage their children to use it [15].

Young individuals consider it as a very popular and acceptable product, thus, subsequently become addicted to it [16] because of arecoline (nicotinic based alkaloid in areca nut) [17, 18] and nicotine in SLT [19]. Once adolescents start consuming SLT, it becomes an essential component of their lives since SLT is the fourth commonly abused substances worldwide and develops dependency like alcohol, nicotine & caffeine with both stimulating and relaxing effects [20–22]. School going children also get fascinated by the undesirable marketing of sparkling packages of SLT products and spend the substantial amount of their pocket money on the purchase of these dangerous substances [23, 24].

A majority of the users get engaged in this habit even before 15 years of age due to influence by the media (40.2%), family and friends’ pressure (30.8%) [25] while in another study, peer pressure (76%) was reported.

Thus, the current survey is to establish the prevalence of smokeless tobacco and/or betel quid in school going adolescents, along with the combined effects of individual, social and environmental factors that influence the use of SLT and/or BQ in them. Previous studies have shown effects of either more individual based influencing factors on SLT and/or BQ use, and have not focused on adolescents [5, 26] or have taken tobacco use in smoked and/or both smoked alongside smokeless forms [27, 28]. This study is one of its kind in the current setting as no such large primary study has focused on the most vulnerable high-risk group for specific SLT and/or BQ use along with the determination of house-level, social, school-level and environmental factors that may influence their use.

## Methods

### Aim

Our purpose of the study was to observe the differential individual, social and environmental features amongst smokeless tobacco and/or Betel quid (SLT and/or BQ) users to determine the key influencers of its use in school going adolescents of Karachi.

### Data source

This study was conducted at government and private schools of Karachi. With a population of approximately 24 million, Karachi is the largest city of Pakistan [29]. Karachi City government has divided the city into six (6) districts in 2013 with each district being further divided into administrative towns of total 18 in number [30].

### Sampling and study participants

Out of six districts, 26 clusters (secondary schools) were selected by systematic random sampling which was proportionate to the number of each school type (ensuring equal participation of government and private schools). The students of 11–16 years of age, studying in grade VI – Grade X were recruited by systematic sampling-kth number. Fifty-100 students per school were included in the study depending on the school and class size from each of the secondary classes that were present on the day of our visit till a desired number was reached.

This therefore resulted in a total sample size of 2200 that may be considered representative of the 11–16 years adolescents of Karachi.

Students of younger or older than 11–16 years, and those who already were undergoing treatments for Oral Cancer were excluded.

### Survey participants

For recruitment purposes, we contacted principals of selected schools (both government and private) to provide them with details regarding the purpose of the study and requested for their inclination to participate in the study. If any selected school refused to participate then another school of same profile was sent an invitation. Schools’ heads were also requested to send consent forms (provided to schools) to the parents along with a covering letter in which they were provided with all relevant details regarding the study and were encouraged to contact principal investigator via a text on given contact number who then called parents to respond to all queries since we did not have toll-free numbers [31]. Parents were requested to sign an acceptance or refusal on the form and sent it back to school by a specific decided date.

### Data collection tool and study variables

We used structured, pre-tested questionnaire (in which few were adapted from GYTS (Global Youth Tobacco Survey) [32]) that gathers the information pertinent to:

*Individual based inquiry* that included information regarding age, sex, parents’ education and occupation, weekly pocket money (this was inquired as, “during an average week, how much money [PKR {USD}] do you have that you can spend on yourself, however you want?” a. 100–200 {0.95–1.90 USD}, b. 201–300 {1.91–2.85 USD}, c. 301–400 {2.86–3.80 USD}, d. 401–500{3.81–4.74 USD} or e. more than 500{4.74 USD}), and the school type (government or private schools).

*Social/home based assessment* that was inclusive of peers’ and parents’ SLT and/or BQ use, teachers’ SLT and/or BQ use status, and SLT and/or BQ use susceptibility if any close friend offers it (this was asked as, “if one of your best friends offered you smokeless tobacco and/or BQ, would you use it?” a. Definitely not. b. Probably not, c. Probably yes, d. Definitely yes).

*School and environment level questions* included availability of SLT and/or BQ at school canteen (the question was, “do you get to buy chewing tobacco, pan, chalia, naswar and gutka in school canteen?” a. Yes, b. No) and with outside hawkers (this was asked as, “do you get to buy chewing tobacco, pan, chalia, naswar and gutka from outside school hawkers?” a. Yes, b. No) and education imparted regarding harmful health effects of SLT and/or BQ use in classroom setting.

Outcome variable was current smokeless tobacco (SLT) and/or betel quid (BQ) use status where user was defined by the use of any or all type of SLT and/or BQ at least once in past 30 days [33] and non-user had not used any or all type of SLT and/or BQ once at least in past 30 days. The students were asked about their BQ and/or SLT consumption by asking:

Do you use any of the following products?

a. Paan (betel quid), b. Paan masala (chalia.betel nut), c. Zarda, d. Ghutka, e. Naswar or f. Others ___________________, please specify.

This was further re-assessed by second series of questions to avoid recall bias like,

1. How many numbers of chews per day you do?
2. Since how many years you have been chewing?
3. What type of SLT/or BQ you chew?

a. Areca nut alone, b. Betel quid without tobacco, c. Betel quid with tobacco, d. Any other type of SLT.

Based on the local school grading hierarchy, mostly less than 12 years old students were housed in lower secondary segment of the school while the older group comprised of higher secondary school stratum.

### Statistical analysis

Data of the current study were analyzed by using Stata v14. Descriptive statistics including mean, frequency and percentages were reported, and Chi-square analysis was performed to determine significance of association between variables and outcome. Associations between the outcome variable “SLT and/or BQ use” and all study variables were assessed by using univariate and multivariate binary logistic regression analyses. Results were reported as both crude form and adjusted odds ratio after adjusting for all remaining individual, social and environmental level variables with 95% confidence level. A *p*-value of <.05 was considered significant for all analyses.

## Results

Overall, 2200 students participated from combined 13 government and 13 private schools of Karachi in this study. School’s response rate was 80%.

Sixty participants’ information was incomplete/missing out of total 2200. The final analysis was based on 2140 participants’ complete responses.

### Individual, social and environmental characteristics of adolescents associated with the SLT and/or BQ use

#### Individual based characteristics

An overall 30 days prevalence of SLT and/or BQ use was estimated to be 42.6% (*n* = 912) of the total sample whereas 57.4% (*n* = 1228) participants did not use it in past 30 days. Amongst users, 30.2% (*n* = 275) aged less than or equal to 12 years while 637 (69.8%) were more than 12 years of age; similar age profiling was evident for non-users. Age was positively associated with the SLT and/or BQ use. Approximately equal proportions of males and females comprised of non-user group while SLT and/or BQ consumer group had more males than females which was significantly associated with the use (χ2 = 16.12, df = 1, *p*-value <0.001). Both parents’ education showed a positive relationship with the consumption of SLT and/or BQ. Most of the respondents got 100–300 PKR (0.95–2.85 USD) as weekly pocket money which was pivotal in SLT and/or BQ chew. A significantly high proportion of users was found in co-education schools irrespective of them being government or private (Table 1).

**Table 1:** Sociodemographic Characteristics of Adolescents regarding SLT and/or BQ Use (*n* = 2140)

	NON-USERS	USERS	chi-square value	*p*-value
Characteristics	*N* (%)	*N* (%)		
Individual characteristics				
Age				
11–12 Years	446 (36.3%)	275 (30.2%)	8.91	0.002
> 12 Years	782 (63.7%)	637 (69.8%)		
Gender				
Female	549 (44.7%)	329 (36.1%)	16.12	< 0.001
Male	679 (55.3%)	583 (63.9%)		
School				
Private	833 (67.8%)	520 (57.0%)	26.33	< 0.001
Government	395 (32.2%)	392 (43.0%)		
Father’s Education				
No formal education	331 (27.0%)	264 (28.9%)	34.39	< 0.001
Primary	165 (13.4%)	182 (20.0%)		
Secondary	422 (34.4%)	318 (34.9%)		
Higher Education	310 (25.2%)	148 (16.2%)		
Mother’s Education				
No formal education	419 (34.1%)	363 (39.8%)	24.35	< 0.001
Primary	197 (16.0%)	173 (19.0%)		
Secondary	408 (33.2%)	284 (31.1%)		
Higher Education	204 (16.6%)	92 (10.1%)		
Parent’s Occupation				
Neither works	61 (5.0%)	49 (5.4%)	0.56	0.907
Only father works	967 (78.7%)	710 (77.9%)		
Only mother works	45 (3.7%)	31 (3.4%)		
Both work	155 (12.6%)	122 (13.4%)		
Weekly pocket money				
< 100	130 (10.6%)	68 (7.5%)	17.18	< 0.001
100–300	727 (59.2%)	618 (67.8%)		
> 300	371 (30.2%)	226 (24.8%)		
School Type-Gender Based				
Only Girls	258 (21.0%)	132 (14.5%)	20.38	< 0.001
Co-education	644 (52.4%)	558 (61.2%)		
Only Boys	326 (26.5%)	222 (24.3%)		

*SLT* Smokeless tobacco, *BQ* Betel quid

Univariate analysis suggested that being a male, government school student, having weekly pocket money more than PKR 300 (2.85 USD) and studying in co-education system were significantly and positively associated with SLT and/or BQ use (Table 3).

Effect sizes (positive association with SLT and/or BQ use) of co-education or only boys’ school adolescents, government school students and children who had a weekly pocket money that they had a luxury of spending the way they want, increased after multivariate adjustments (Table 3).

#### Social characteristics

The use of ST and/or BQ by adolescents was positively linked with its use by their peers (χ2 = 2.29, df = 3, *p*-value <0.001) and parents (χ2 = 64.73, df = 2, *p*-value <0.001) (Table 2).

**Table 2:** Social & Environmental Characteristics of Adolescents regarding SLT and/or BQ Use (n = 2140)

	NON-USERS	USERS	chi-square value	*p*-value
Characteristics	*N* (%)	*N* (%)		
Social Characteristics				
SLT and/or BQ use by peers				
None of them uses it	631 (51.4%)	201 (22.0%)	2.29	< 0.001
Some of them use it	411 (33.5%)	370 (40.6%)		
Most of them use it	137 (11.2%)	235 (25.8%)		
All of them use it	49 (4.0%)	106 (11.6%)		
SLT and/or BQ use by teachers				
None of them uses it	852 (69.4%)	606 (66.4%)	4.87	0.182
Some of them use it	278 (22.6%)	236 (25.9%)		
Most of them use it	97 (7.9%)	67 (7.3%)		
All of them use it	1 (0.1%)	3 (0.3%)		
SLT and/or BQ use by parents				
No one uses it	980 (79.8%)	586 (64.3%)	64.728	< 0.001
any one of them uses it	226 (18.4%)	293 (32.1%)		
Both of them use it	22 (1.8%)	33 (3.6%)		
Use susceptibility if closest friend offers it				
Definitely Not	997 (81.2%)	465 (51.0%)	2.93	< 0.001
Probably No	127 (10.3%)	98 (10.7%)		
Probably Yes	53 (4.3%)	133 (14.6%)		
Definitely Yes	51 (4.2%)	216 (23.7%)		
Environmental Characteristics				
Class discussion in past 12 months regarding SLT and/or BQ use				
Yes	612 (49.8%)	378 (41.4%)	15.04	0.001
No	597 (48.6%)	520 (57.0%)		
Not sure	19 (1.5%)	14 (1.5%)		
SLT and/or BQ available at school canteen				
No	1121 (91.3%)	686 (75.2%)		
Yes	107 (8.7%)	226 (24.8%)	1.03	< 0.001
SLT and/or BQ available outside school				
No	1084 (88.3%)	508 (55.7%)		
Yes	144(11.7%)	404 (44.3%)	2.92	< 0.001

*SLT* Smokeless tobacco, *BQ* Betel quid

Univariate analysis indicated that adolescents whose peers used SLT and/or BQ had 6.8 times higher odds of consuming it as compared with those who did not have user peers (Table 3) and this remained significant after adjusting for SLT and/or BQ use by parents, teachers, use susceptibility, individual and environmental characteristics in a multivariate model though its effect size reduced (OR = 2.68, 95% CI 1.71–4.19, *p*-value <0.001) (Table 3). Either of the parents’ use of SLT and/or BQ also encouraged use in adolescents as indicated by both univariate and multivariate analyses (Table 3).

**Table 3:** Sociodemographic, Social & Environmental Characteristics of Adolescents associated with SLT and/or BQ Use (n = 2140)

SLT and/or BQ Users				
	OR (95% CI)	*p*-value	aOR (95% CI)	*p*-value
Characteristics				
Individual characteristics				
Age				
11–12 Years	1		1	
> 12 Years	1.32 (1.10–1.58)	*0.003*	1.00 (0.80–1.25)	0.991
Gender				
Female	1		1	
Male	1.43 (1.20–1.70)	*< 0.001*	1.38 (1.05–1.83)	*0.020*
School				
Private	1		1	
Government	1.59 (1.33–1.89)	*< 0.001*	2.06 (1.49–2.85)	*< 0.001*
Father’s Education				
No formal education	1		1	
Primary	1.38 (1.06–1.80)	*0.017*	0.96 (0.68–1.36)	0.860
Secondary	0.94 (0.76–1.17)	0.609	0.86 (0.65–1.14)	0.314
Higher Education	0.59 (0.46–0.77)	*< 0.001*	0.96 (0.68–1.34)	0.816
Mother’s Education				
No formal education	1		1	
Primary	1.01 (0.79–1.29)	0.915	1.01 (0.73–1.39)	0.927
Secondary	0.80 (0.65–0.98)	*0.038*	0.93 (0.71–1.22)	0.614
Higher Education	0.52 (0.39–0.69)	*< 0.001*	0.89 (0.61–1.30)	0.897
Parent’s Occupation				
Neither works	1		1	
Only father works	1.02 (0.65–1.59)	0.929	1.15 (0.72–1.84)	0.540
Only mother works	0.93 (0.72–1.20)	0.595	1.22 (0.61–2.43)	0.572
Both work	0.87 (0.52–1.46)	0.612	1.33 (0.78–2.26)	0.291
Weekly pocket money				
< 100	1		1	
100–300	0.85 (0.61–1.20)	0.375	1.51 (1.03–2.22)	*0.032*
> 300	1.39(1.14–1.70)	*0.001*	1.17 (0.77–1.79)	0.445
School Type-Gender Based				
Only Girls	1		1	
Co-education	1.69 (1.34–2.15)	*< 0.001*	2.88 (1.89–4.40)	*< 0.001*
Only Boys	1.33 (1.01–1.74)	*0.038*	2.00 (1.27–3.14)	*0.003*
Social Characteristics				
SLT and/or BQ use by peers				
None of them uses it	1		1	
Some of them use it	2.82 (2.28–3.49)	*< 0.001*	2.07 (1.63–2.63)	*< 0.001*
Most of them use it	5.38 (4.13–7.01)	*< 0.001*	3.02 (2.23–4.09)	*< 0.001*
All of them use it	6.79 (4.67–9.87)	*< 0.001*	2.68 (1.71–4.19)	*< 0.001*
SLT and/or BQ use by teachers				
None of them uses it	1		1	
Some of them use it	1.19 (0.97–1.46)	0.807	1.18 (0.93–1.51)	0.157
Most of them use it	0.97 (0.69–1.34)	0.861	0.91 (0.61–1.36)	0.676
All of them use it	4.21 (0.43–40.64)	0.213	1.29 (0.11–14.64)	0.833
SLT and/or BQ use by parents				
No one uses it	1		1	
any one of them uses it	2.16 (1.73–2.65)	*< 0.001*	1.52 (1.19–1.94)	*0.001*
Both of them use it	2.50 (1.44–4.34)	*0.001*	1.42 (0.71–2.82)	0.314
Use susceptibility if closest friend offers it				
Definitely Not	1		1	
Probably No	1.65 (1.24–2.20)	*0.001*	1.36 (0.98–1.89)	0.059
Probably Yes	5.38 (3.84–7.53)	*< 0.001*	3.36 (2.30–4.90)	*< 0.001*
Definitely Yes	9.08 (6.56–12.56)	*< 0.001*	4.47 (3.10–6.44)	*< 0.001*
Environmental Characteristics				
Class discussion in past 12 months regarding SLT and/or BQ use				
Yes	1		1	
No	1.41 (1.18–1.67)	*< 0.001*	1.25 (1.01–1.55)	*0.033*
Not sure	1.19 (0.59–2.40)	0.622	1.09 (0.47–2.50)	0.831
SLT and/or BQ available at school canteen				
No	1		1	
Yes	3.45 (2.69–4.42)	*< 0.001*	1.29 (0.94–1.76)	0.107
SLT and/or BQ available outside school				
No	1		1	
Yes	5.98 (4.81–7.44)	*< 0.001*	3.65 (2.82–4.73)	*< 0.001*

*SLT* Smokeless tobacco, *BQ* Betel quid, *OR* Odds ratio, *aOR* adjusted odds ratio, *CI* Confidence intervals, Significant results are italicized.

The teenagers who will accept the offer of using this product by their friends, were 9 times more likely to use it as compared with those who would not accept it, though this association attenuated but remained statistically significant after adjusting for multiple covariates (OR = 4.47, 95% CI 3.10–6.44, *p*-value <0.001) (Table 3).

#### Environmental characteristics

Adolescents who did not have knowledge regarding harmful health effects of SLT and/or BQ were significant users of SLT and/or BQ (χ2 = 15.04, df = 2, *p*-value 0.001) than those who attended awareness sessions (Table 2). Multivariate analysis indicated that there are 25% more chances of SLT and/or BQ use if little or no awareness was created in class sessions (Table 3).

Availability of SLT and/or BQ at school canteen and with outside school hawkers (χ2 = 2.92, df = 1, *p*-value 0.001) played a sizeable role in the use (Table 2). The users were 6 times more likely to use SLT and/or BQ if it was available with outside school hawker relative to when it was not accessible with outside school vendors, this was persistently indicated by both univariate (OR = 5.98, 95% CI 4.81–7.44, *p*-value <0.001) and multivariate analyses (OR = 3.65, 95% CI 2.82–4.73, *p*-value <0.001) (Table 3) after adjusting for other environmental, individual and social determinants for SLT and/or BQ use.

## Discussion

In this study, we found that male students studying either in co-education or boys school, schools where availability of these products was substantial with an easy access, adolescents having peers, friends and parents using these products, and if there was less or no awareness provided at school regarding ill-health effects of SLT and/or BQ; they all set a conducive environment for more provocative use of SLT and/or BQ consumption.

Overall, the prevalence of SLT and/or BQ in our study (42.6%) was lower than that found in another study which also included smoking and alcohol consumption (72.5%) [26]. We included only different types SLT and/or BQ as these are more pertinent determinants of oral potentially malignant lesions and oral cancer in South East Asia including Pakistan(10, 15). Moreover, the products we included are more culturally acceptable and easily accessible to this high-risk adolescent group [1, 23, 34].

Our results based on individual factors prompting use, revealed that adolescents studying at government schools and male gender were more prone to the substance abuse as seen in other studies as well [34–37]. The use of SLT and/or BQ by males was a more prominent finding in contrast to the conclusion of South Asian betel-quid consortium where females were profound users as they were chronic users with low educational and awareness levels [38]. Whereas, in our present study we had adolescent females who had high awareness, were educated and were comparatively less chronic users.

Co-education schools have been shown to be a substantially significant contributing factor in the consumption of the SLT and/or BQ. This may be better understood if cultural backdrop is kept in forefront that by using these products, boys get more opportunity to impress girls since Pakistani cultural gives less or no freedom to interact and socialize with opposite gender as also seen in other cultures [28, 34, 38]. It is also a well-established fact that adolescents tend to use tobacco as a symbol of maturity and appeal which is provoked by the presence of the opposite gender [34, 39]. Moreover, parents highly discourage their children interacting with opposite gender outside school hours probably due to the fear that kids may get distracted and get low scores in exams or may engage in sexual impropriety [40]. This could possibly explain the greater use of SLT and/or BQ among adolescents in the co-education system.

In our study, parents’ higher education was evidently and inversely related to the probability of BQ/or SLT consumption as it can be anticipated and is also in agreement with other studies [27, 28],

Weekly pocket money which children could spend any way they wanted, proved to be an independent element of this product’s use even after controlling for other confounders. This was not a very robust factor in our study as in other studies [28, 34, 41], despite of the rationale being mentioned elsewhere that these cheaper products are readily available as compared with other similar consumables.

Social factors like, peer pressure, use of the product by friend or either of the parents were the prevalent and significant contributors towards SLT and/or BQ use in our study. This finding along with ‘curiosity to use’ were major determinants in other studies that focused more on tobacco use and less on SLT and/or BQ thus augmenting the need of interventions aimed more towards these communal influences [34, 42–44]. Together with, susceptibility of indulging in SLT and/or BQ chew becomes highly substantial if any close friend offers it; this statistically significant finding of our study was coherent with other studies conducted elsewhere [27, 34, 44–47].

Our results suggested that knowledge regarding ill-effects of substance abuse played a vital role in limiting the habit of all addictions including smoking, smokeless tobacco, alcohol consumption and areca nut consumption; this finding was in coherence with other studies [38, 48]. If class awareness sessions regarding SLT and/or BQ hazardous effects were not held in schools then there was 41% more likelihood of its consumption amongst users as compared with when they were frequent feature. Also, if these sessions are regularly held at schools, then there is a high probability to revert the “coolness” effect from use to non-use; where adolescents may refrain from its consumption in front of opposite gender to display maturity and maintain attractiveness.

Moreover, our study revealed that the easy availability of the product outside the school premises and sparklingly marketed products also play a significant role in the use as seen in other studies as well [25, 34].

Future interventions may be geared towards three major elements that can promise positive results in curbing the SLT and/or BQ use. Based on the results of the current study, these may include interventions focusing on awareness sessions on SLT and/or BQ harmful health effects, peer and friends pressure on use, and the products’ availability outside schools. These results were inferred more robustly from this study as it was primarily aimed towards SLT and/or BQ unlike other studies that considered all forms of tobacco while SLT and/or BQ being just a small component of it [34, 42–44].

Limitations of the current study included firstly; as it is a self-reported data, it may have compromised the quality due to under reporting, though participation was completely voluntary with consent and participants were assured that anonymity of the information provided will be maintained. Secondly, as with all similar studies, bias in recall must be considered while interpreting data which was also regulated by prompting and asking questions in different ways to keep a counter check, for example, for outcome variable some students denied when they were asked about consumption of SLT and/or BQ but then in the next section, did describe the type of SLT and/or BQ used by them. Though the use was not validated with any hard outcome, yet it was ascertained by multiple questions. Thirdly, since it is a cross sectional study thus causality must be ascertained by other rigorous methods. Lastly, there may be un-known confounders that we did not control in our analysis.

Fortes of the current study can be emphasized by being first large representative sample based study to the best of our knowledge to assess risk behaviors in the form of combined effects of individual, social and environmental factors that promote/influence use of SLT and/or BQ in a high-risk group of school-going adolescents in Karachi. Approximately equal representation of both genders from both type of schools that is government and private, was again a highlighted feature of current study thus overall prevalence of use alongside, prevalence amongst each gender group was ascertained. Another accentuated feature of the contemporary study was getting information on most behaviors associated with SLT and/or BQ use amongst adolescents inclusive of all most probable individual, social and environmental contributors.

## Conclusion

In summary, a co-education or boys’ school male student, with influencers in the form of parents, friends and peers, lack of awareness regarding SLT and/or BQ undesirable ill-health effects, and availability of the cheap product with hawkers outside school; all contribute positively towards enhanced risk of substance abuse in adolescents. These associations were very strongly related with more use. School based interventions which focus on these contributors might bring out positive results in curbing the habit and as a result reducing the disease burden.

## Abbreviations

BQ: Betel Quid
PKR: Pakistani currency
SLT: Smokeless Tobacco
